# dbAQP-SNP: a database of missense single-nucleotide polymorphisms in human aquaporins

**DOI:** 10.1093/database/baad012

**Published:** 2023-03-13

**Authors:** Rachana Dande, Ramasubbu Sankararamakrishnan

**Affiliations:** Department of Biological Sciences and Bioengineering, Indian Institute of Technology Kanpur, Kanpur, Uttar Pradesh 208016, India; Department of Biological Sciences and Bioengineering, Indian Institute of Technology Kanpur, Kanpur, Uttar Pradesh 208016, India; Mehta Family Centre for Engineering in Medicine, Indian Institute of Technology Kanpur, Kanpur, Uttar Pradesh 208026, India

## Abstract

Aquaporins and aquaglyceroporins belong to the superfamily of major intrinsic proteins (MIPs), and they transport water and other neutral solutes such as glycerol. These channel proteins are involved in vital physiological processes and are implicated in several human diseases. Experimentally determined structures of MIPs from diverse organisms reveal a unique hour-glass fold with six transmembrane helices and two half-helices. MIP channels have two constrictions formed by Asn-Pro-Ala (NPA) motifs and aromatic/arginine selectivity filters (Ar/R SFs). Several reports have found associations among single-nucleotide polymorphisms (SNPs) in human aquaporins (AQPs) with diseases in specific populations. In this study, we have compiled 2798 SNPs that give rise to missense mutations in 13 human AQPs. To understand the nature of missense substitutions, we have systematically analyzed the pattern of substitutions. We found several examples in which substitutions could be considered as non-conservative that include small to big or hydrophobic to charged residues. We also analyzed these substitutions in the context of structure. We have identified SNPs that occur in NPA motifs or Ar/R SFs, and they will most certainly disrupt the structure and/or transport properties of human AQPs. We found 22 examples in which missense SNP substitutions that are mostly non-conservative in nature have given rise to pathogenic conditions as found in the Online Mendelian Inheritance in Man database. It is most likely that not all missense SNPs in human AQPs will result in diseases. However, understanding the effect of missense SNPs on the structure and function of human AQPs is important. In this direction, we have developed a database dbAQP-SNP that contains information about all 2798 SNPs. This database has several features and search options that can help the user to find SNPs in specific positions of human AQPs including the functionally and/or structurally important regions. dbAQP-SNP (http://bioinfo.iitk.ac.in/dbAQP-SNP) is freely available to the academic community.

**Database URL** http://bioinfo.iitk.ac.in/dbAQP-SNP

## Introduction

**Figure 1.**
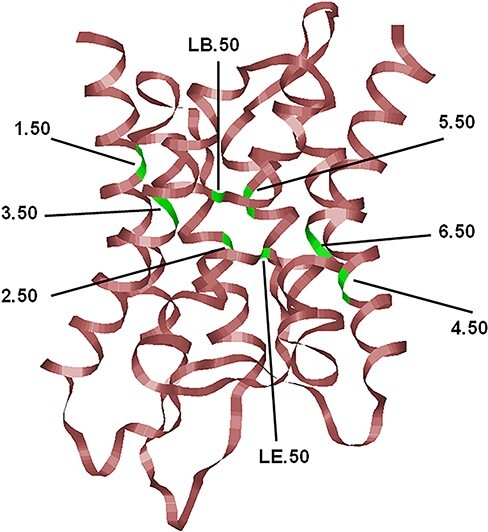
The typical hour-glass fold adopted by MIP family members shown here corresponds to the human AQP4 (PDB ID: 3GD8; resolution: 1.8 Å). Only the backbone is shown in ribbon representation. The most conserved residue in each TM segment (TM1–TM6) and the two half-helices (LB and LE) are displayed and the locations of the conserved residues are indicated, and their generic numbers are also shown. In human AQP4, they correspond to E44 (1.50), G78 (2.50), N97 (LB.50), Q122 (3.50), E163 (4.50), G194 (5.50), N213 (LE.50) and P237 (6.50), and the residue numbering corresponds to the PDB ID: 3GD8. For details about the generic numbering scheme in AQPs, see the main text.

Members of the superfamily of major intrinsic proteins (MIPs) can be found in all three kingdoms of life (1–3). In some species groups like plants and fungi, MIP homologs are present abundantly and in multiple numbers (4–6). In humans, 13 MIP homologs are present. Since aquaporin (AQP) is a prototype member of this family, these proteins are named as AQP0 to AQP12, and hence these human MIP homologs will be referred to as human AQPs hereafter. Sequence analyses and phylogenetic studies reveal that the human AQPs can be broadly classified into three classes (3, 7, 8). Those which specifically transport water (AQP0, AQP1, AQP2, AQP4, AQP5, AQP6 and AQP8) belong to classical AQPs. The human MIP homologs that prefer to transport glycerol and other neutral solutes fall under the category of aquaglyceroporins (AQP3, AQP7, AQP9 and AQP10). AQP homologs belonging to the third category are called ‘Super AQPs’ (AQP11 and AQP12). MIPs are one of the well-characterized membrane protein families, and the structures of several MIPs have been determined from different species groups. Despite low sequence identity, MIPs from bacteria, yeast, plants and mammals adopt a unique hour-glass helical fold (Figure 1) with six transmembrane (TM) helices (TM1–TM6) and two half-helices (LB and LE) (1). Two-fold rotational pseudo-symmetry is observed in the structure which is also reflected in MIP sequences. The two halves of an MIP sequence exhibit sequence similarity indicating gene duplication and fusion during evolution. The sequence motif Asn-Pro-Ala (NPA) is predominantly conserved in the majority of MIP members in both the half-helices LB and LE present, respectively, in the N- and C-terminal halves. The channel is also characterized by a narrow constriction formed by four residues called the aromatic/arginine selectivity filter (Ar/R SF). Experimental and computational studies demonstrate that both NPA motifs and Ar/R SFs have an important role in the transport and selectivity of solutes that are transported across the membrane (9–13). Residues near the NPA motif exhibit characteristic conservation in AQPs and aquaglyceroporins (14), and simulation studies reveal that specific interactions involving these residues could play important roles in the transport properties of MIP channels (15). AQP homologs form tetramers under physiological conditions with each monomer having a functional aqueous pore (16).

Point mutations in human AQP homologs are known to cause various diseases and result in abnormal water homeostasis. The range of defects due to genetic variants includes misfolding, problems in tetramer assembly, failure to transport the substrates or protein targeting/sorting (17). In this regard, single-nucleotide polymorphisms (SNPs) in several human AQPs have been investigated, and their associations with several diseases have been studied in specific populations. Several reports suggest an association among SNPs of classical AQPs and diseases and/or important physiological processes. SNPs of *AQP1* and other genes were found to have an association in male patients with a history of priapism indicating AQP1’s involvement in important cellular processes such as cell adhesion and cell signaling (18). A study involving male long-distance runners found that a genetic variant of AQP1 was found to be associated with acute body fluid loss (19). The involvement of AQP1 polymorphisms has been shown to be important in water retention among patients with liver cirrhosis (20). Wang *et al.* genotyped AQP2 and AQP9 polymorphisms in lung cancer patients and showed that they contribute to chemotherapy response (21). Genotyping 10 polymorphisms of salivary samples from ∼700 individuals found an association among *AQP1*, *AQP2*, *AQP5* and *AQP6* genes and periodontitis and temporomandibular joint disorders (22). Genotyping of seven AQP3 SNPs in early breast cancer patients indicated that AQP3 could be a potential prognostic marker (23). Using water permeability assays, Sorani *et al.* have shown that four non-synonymous SNPs (nsSNPs) in AQP4-reduced water permeability (24). With AQP4 playing a crucial role in maintaining the brain water balance, the authors suggested that these nsSNPs may have a significant role in diseases such as cerebral edema. AQP4 SNPs have also been associated with sleep quality, latency and duration, which suggested a relationship between sleep and brain Aβ-amyloid burden (25). Larsen *et al.* established a link between AQP4 SNPs and non-rapid eye movement sleep (26). The association of AQP4 SNPs with serum S100 calcium-binding protein B and schizophrenia has been investigated by Wu *et al.* (27). The association of AQP4 SNPs has been investigated in sudden infant death syndrome, neuromyelitis optica, vascular depression, schizophrenia and intracerebral hemorrhage (28–32). Polymorphism in the *AQP5* gene has been shown to have an association with a reduced risk of chronic obstructive pulmonary disease in the Chinese Han and European American populations (33, 34). SNPs in AQP5 indicate that *AQP5* and other genes are involved in the pathogenesis of caries (35, 36). In studies conducted in patients with sepsis and early-stage breast cancer, the AQP5 promoter polymorphism was found to be associated with susceptibility to major adverse kidney events and progesterone receptor positivity, respectively (37, 38).

Polymorphisms in aquaglyceroporins have been shown to have a role in diseases such as Type 2 diabetes and hypertension. SNPs in the *AQP7* gene have been shown to have an association with obesity and Type 2 diabetes in the Caucasian and Chinese Han populations (39, 40). AQP7 SNPs have been shown to be involved in the risk of stroke in patients with hypertension (41). The possible role of AQP8 SNPs has been suggested in the pathogenesis of polycystic ovary syndrome (42). Studies on Thai postmenopausal women revealed an association of AQP9 SNPs with femoral neck bone mineral density (43). The role of AQP4 and AQP9 SNPs in methylation of inorganic arsenic has been studied in Croatian–Slovenian pregnant and non-pregnant women (44). Not much is known about the SNPs of the so-called Super AQPs.

Association studies of human AQP SNPs with several diseases and specific phenotypes are scattered in the literature. The database of SNP (dbSNP) has revealed that many hundreds of SNPs are found in human AQPs (45). We have systematically analyzed the dbSNP and classified the SNPs in human AQP homologs according to their positions in the AQP structure, the nature of amino acid substitutions and the possible disease association. We have compiled these data in the form of a database (dbAQP-SNP) and made this resource freely available in the form of a database. This database will help to look for specific SNPs in human AQP homologs and aid in experimental design to understand the effect of SNPs on the structure and function of human AQPs.

## Materials and methods

Human reference genome sequences and reference protein sequences available from the RefSeq database (46) are used to identify the variations, respectively, in the human genome and the corresponding protein sequences. Although SNPs can be of different types, we only considered the missense variants in human AQPs, AQP0–AQP12. The dbSNP as updated in May 2019 was queried to collect and compile all the SNPs of human AQPs. The typical query used in the dbSNP was ‘aqp1’ (Gene Name) AND ‘missense variant’ (Function Class). Each dbSNP entry is assigned a unique Accession ID called Reference SNP ID (rsID) and contains information regarding the variation in the nucleotide, codon and the resulting change in the amino acid of the protein with respect to the reference sequence. We have used only the primary isoforms of human AQPs for determining the variations in the protein sequences (Supplementary Table S1). The predicted and minor isoforms were not considered. The search results were downloaded as a batch JavaScript Object Notation (JSON) file. For ease of further analysis, the data were processed into comma-separated values (CSV) files using the JSON module in Python. We removed the duplicates and the deprecated files in the search results. Similarly, we excluded results that contained non-sense and synonymous variations.

### Construction of the dbAQP-SNP database

The contents of the database containing information regarding SNPs of human AQPs are stored as CSV files since they are easy to store and upgrade. The website is maintained on an Apache HTTP server v2.4 (https://httpd.apache.org). The webpages are developed in HTML 5, and the JavaScript used is loaded through a content delivery network. Python Common Gateway Interface (CGI) scripts are used for dynamic webpages which are generated according to the user’s input. CGI modules are used to communicate between the webpages and the Python scripts at the backend. When the user submits a query, a CGI script is invoked which processes the query and generates an HTML response for the user to view.

Positions of the residues that are changed due to missense variants were visualized in the respective experimentally determined structures or models. The 3D representation of the residue under consideration in the structure was implemented using NGL viewer (47) embedded as JavaScript. The structure files in the Protein Data Bank (PDB) format for each residue variation, with the side chains of the variant and the reference residue, were generated using the ‘swapaa’ option available in UCSF Chimera v 1.14 (48). For the side chains displayed in the edited structure, the C^α^ coordinates remain the same as that of the reference residue, and the rotamers for the rest of the side chain were generated using the Dunbrack library (49) available in UCSF Chimera.

## Results

We searched the dbSNP for missense variants of human AQPs as described in the Materials and methods section. Our search yielded 2798 SNPs that resulted in missense variations that are found in 13 human AQP homologs. The number of entries found varied across different human AQPs, from 173 to 353. Among AQP0–AQP12 homologs, AQP7 and AQP0 have the largest and least numbers of entries, respectively. These data have been organized into a database called dbAQP-SNP available freely at http://bioinfo.iitk.ac.in/dbAQP-SNP. We first describe the salient features of the database and then present an analysis of the SNPs found in the human AQP homologs.

### dbAQP-SNP database

**Figure 2.**
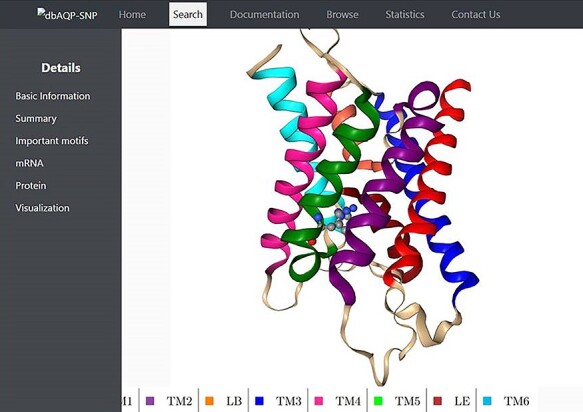
Molecular plot of a specific human AQP with the residue in which missense SNPs occurred shown in ball-and-stick representation. The helices (TM1–TM6) and the half-helices (LB and LE) are displayed in different colors.

We have compiled the details of missense mutations for the 2798 SNPs of 13 human AQP homologs, and the details of all these entries are available in the dbAQP-SNP database. The database is organized into different sections. Each entry has a unique ID along with the reference SNP ID (rsID). The page for each entry has several details such as variation in the codon, the residue number and the TM segment/loop in which it occurs, the original residue and the mutated residue as the result of SNP, details of NPA motifs and Ar/R SFs, the position of the codon at which the SNP occurs, the original codon and the codon modified due to SNP and the generic number of the residue which is substituted due to missense variation. The molecular plot of a human AQP with the substituted residue due to missense SNPs from a sample entry in the dbAQP-SNP database is shown in Figure 2.

### Different search options

**Figure 3.**
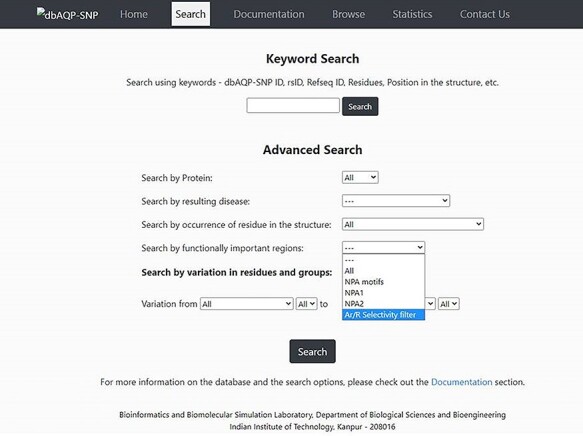
Screenshot of the dbAQP-SNP Search page.

The dbAQP-SNP database provides various search options (Keyword Search and Advanced Search) for the users to easily navigate the database. With the Keyword Search, the user can search the database using the unique dbAQP-SNP ID, rsID or RefSeq ID. In the ‘Advanced Search’ option, the user can provide multiple parameters to search at the same time by combining all the terms. For example, the user can search by protein, and this will help to find all SNPs associated with a specific human AQP homolog. One can also search the database for SNPs associated with a specific disease. The AQP structure is divided into many regions such as channel-facing and helix–helix interface (explained in the section “Substitutions due to SNPs in the context of structurally and/or functionally important regions”). The user can search all the SNPs that are found in specific structural regions of AQPs. As the NPA motif and Ar/R SF have been shown to be functionally important, a search option is available that will fetch all the SNPs that result in missense variation in these functionally important regions. The amino acids are divided into six groups (see the section “Pattern of missense SNP substitutions”) for the purpose of understanding the pattern of substitution. A search option is also implemented in which the user can get all the SNPs which results in the substitution of amino acids belonging to one of the six groups to any of the six groups. This will also help to find non-conventional substitutions such as hydrophobic to charged or small residues to aromatic residues. These different search options can also be combined in the ‘Advanced Search’ option. For example, the user can search for all SNPs found in AQP0 that occur in the helix–helix interface in which charged residues are substituted by aromatic residues. The screenshot of the dbAQP-SNP Search page is shown in Figure 3.

### Documentation and statistics page

The ‘Documentation’ page provides a brief introduction to human AQPs and explains the database, different search options, classification of amino acids and structural regions of AQP channels. The Statistics page presents the statistics for each of the 13 human AQPs and the occurrence of SNPs for each human AQP in different structural regions such as TM region, channel-facing, helix–helix interface, monomer–monomer interface and lipid-facing. The statistical data for all the human AQPs in which SNPs occur in the cytoplasmic, extracellular, loops and N- or C-terminal regions can also be found on the same page.

Overall, this will be a useful resource where SNPs in different human AQPs are compiled and this resource will be helpful to predict the missense variations that are likely to disrupt the structure and function and the hypothesis generated from this study can be tested experimentally. In the following sections, we have analyzed the data available in the database and looked into the pattern of amino acid substitutions due to missense SNPs and substitutions that occur in the structurally and/or functionally important regions such as NPA motifs, Ar/R SFs and helix–helix interfaces. The likely influence of unusual substitutions in the context of the function is also discussed.

### Pathogenicity due to SNPs in human AQPs

A missense variation can be pathogenic and can cause many types of diseases. A missense mutation can be deleterious if it results in ‘a genetic alteration that increases an individual’s susceptibility or predisposition to a certain disease or disorder’ (https://www.cancer.gov/publications/dictionaries/genetics-dictionary). The disease condition could be the result of loss/gain of function. Two such examples are Gly^5.60^ → Arg in AQP2 and Ile^5.61^ → Phe in AQP5 (50, 51). Improper trafficking is another factor that will result in disease conditions. In the case of AQP2, a Glu residue at the C-terminus is replaced by Lys and this substitution results in protein being retained in the Golgi complex and the protein fails to reach the plasma membrane (52). We have used the Online Mendelian Inheritance in Man (OMIM) database (http://www.omim.org) (53) and ClinVar available at the National Center for Biotechnology Information (www.ncbi.nlm.nih.gov/clinvar) (54) to ascertain the pathogenicity of SNPs in human AQPs in the current dataset. We have found 22 SNPs that are reported to be implicated in disease conditions, and they mainly occur in AQP0, AQP2 and AQP5. These diseases include cataract (AQP0), autosomal recessive nephrogenic diabetes insipidus (AQP1 and AQP2) and palmoplantar keratoderma (AQP5) and diminished glycerol release (AQP7). Most of the missense mutations in these cases involve non-conservative substitutions such as Glu → Gly, Thr → Arg, Pro → Leu, Gln → Pro, Thr → Met, Gly → Arg, Arg → Cys, Glu → Lys and Ala → Glu.

### Pattern of missense SNP substitutions

It is clear that there are examples of human AQPs in which non-conservative substitutions due to missense SNPs give rise to pathogenic conditions. These are only a small fraction of all known missense SNPs in human AQPs. With 2978 examples in human AQPs, we first wanted to find out the nature of amino acid substitutions as a result of missense variations. For this purpose, the amino acids were classified into six groups. The residues Gly, Ala, Thr, Ser and Cys, collectively grouped as small and weakly polar (SWP) residues, have been shown to be highly group conserved at the helix–helix interface of many TM helical proteins including AQPs (55–57). The other groups are aliphatic-hydrophobic (AH; Leu, Ile, Val and Met), aromatic (Phe, Tyr and Trp), charged (Lys, Arg, His, Asp and Glu) and neutral polar (NP; Asn and Gln). The sixth group has only one residue Pro. Proline is known to introduce kink in α-helices (58). Hence, we considered Pro separately and did not group into any of the categories mentioned above. Such grouping of amino acids has been previously used in other studies (59, 60).

Our analysis has revealed some interesting observations. Table 1 summarizes the number of substitutions involving amino acids of all six groups. It shows that the highest number of substitutions involves the group of residues that are classified as SWP residues. There are 1086 examples in which SWP residues are substituted by another member of this group (427) or any residue from the other five groups (659). We found 73 and 50 cases in which SWP residues are substituted by larger aromatic or proline residues, respectively. We found a significantly larger number of 242 examples that involve substitutions from SWP residues to charged residues. Surprisingly, very few examples (26) are found in which SWP residues are substituted by NP residues. As far as the individual residues within the SWP group are concerned, the maximum number of substitutions involved Ala (Ala-Thr: 125; Ala-Val: 115) and Gly (Gly-Ser: 72; Gly-Arg: 88).

**Table 1.** Pattern of missense SNP substitutions in human AQP homologs

Group^a^^,^^b^	SWP	AH	Charged	NP	Aromatic	Pro	Total
SWP	427	268	242	26	73	50	1086
AH	188	457	55	18	79	60	857
Charged	116	30	182	120	62	28	538
NP	38	10	70	0	0	15	133
Aromatic	78	70	32	8	8	0	196
Pro	80	57	27	4	0	0	168
Total	927	893	610	176	222	153	2978

a For each amino acid group shown in the left column, the substitutions of amino acids due to missense SNP mutations resulting in amino acids belonging to the same or different group are shown in the respective row.

b SWP (Gly, Ala, Cys, Ser and Thr); AH (Leu, Ile, Val and Met); Charged: basic and acidic residues (Asp, Glu, Lys, Arg and His); NP (Asn and Gln); Aromatic: aromatic residues (Phe, Trp and Tyr); Pro: Proline.

In the case of the AH group, the largest number of substitutions involves residues within the same group. There are 457 instances in which one residue from the AH group is substituted by another residue within the same group. AH residues due to SNPs are substituted to SWP residues in a larger number compared to any other group, and 188 AH to SWP substitutions constitute the second largest number of missense substitutions. Among the residues in the AH group, Leu-Phe: 56, Leu-Pro: 60, Ile-Thr: 61 and Met-Thr: 31 are some of the substitutions in which AH residues are replaced by residues from other groups.

When it comes to charged residues, maximum substitutions due to SNPs occur (182) within the same group. A significant number of substitutions occur between the charged residues and residues from the SWP (116) or NP (120) groups. Some of the notable substitutions in this group involve Glu (Glu-Lys: 37), Arg (Arg-Cys: 42; Arg-His: 42; Arg-Gln: 45) and His (His-Tyr: 22). The residues in the NP group are mostly substituted by residues from the charged group. The number of instances of Asn substituted by Ser from the SWP group is 30. There is not a single substitution of NP residues to aromatic residues.

As far as the residues in the aromatic group are concerned, Phe-Ser and Phe-Leu have the most number of 22 and 47 substitutions, respectively. The maximum number of substitutions involving proline includes Pro-Leu: 57 and Pro-Ser: 45. However, we have not found a single example in which aromatic residues are substituted by proline or vice versa. Thus substitutions due to SNPs involve small to big, neutral to charged, positively charged to negatively charged or hydrophobic to proline, and vice versa. Such substitutions are not generally considered as conservative substitutions. It would be interesting to see where exactly these substitutions occur in the 3D structure of human AQPs. This knowledge can help to predict whether such substitutions are likely to disrupt the structure and/or function.

### Substitutions due to SNPs in the context of structurally and/or functionally important regions

Structures of 6 out of 13 human AQPs have been determined experimentally, and they all have the characteristic hour-glass helical fold. We have examined the site of missense mutations by mapping them onto the structures. AQP structures from diverse organisms, including *Eschericha coli*, plants and mammals, have been determined experimentally (1). Although these are sequentially distantly related, they all adopt a unique hour-glass helical fold. In the present study, we used the experimentally determined structures for the human homologs AQP1, AQP2, AQP4, AQP5, AQP7 and AQP10. Their respective PDB (61) IDs are 4CSK, 4NEF, 3GD8, 3D9S, 6QZI and 6F7H. For all other human AQPs, we downloaded the modeled structures from the MIPModDB database (http://bioinfo.iitk.ac.in/MIPModDB) developed in our laboratory (62). The protocol used to build these models is described in detail in previous publications (1, 5, 55). Since AQP11 and AQP12 are the most distantly related homologs, we compared the respective models downloaded from MIPModDB with the models predicted using AlphaFold (63, 64) which is based on a machine-learning approach. Superposition of the two models for both AQP11 and AQP12 indicates that the distantly related AQP11 and AQP12 also adopt the helical hour-glass fold (Supplementary Figure S1) and the differences are mainly found in the loop regions connecting the TM segments. The root-mean-square deviation (RMSD) of MIPModDB- and AlphaFold-modeled structures was calculated using ChimeraX (65). The RMSD of AQP11 structures for 129 pruned atom pairs for AlphaFold and MIPModDB models is only 1.004 Å. The RMSD of AQP12 models derived from AlphaFold and downloaded from MIPModDB is 0.986 Å for 120 pruned atom pairs. In calculating the RMSD for pruned atom pairs, the conformationally dissimilar regions such as loop regions are excluded. This has clearly increased our confidence levels in the models available in the MIPModDB database.

There are also examples in which SNPs occurred at the N- or C-terminal regions which were not defined in the experimentally determined structures or were not included in the models. We found 607 SNPs in the N- or C- terminal ends and 474 of them could not be mapped on the structures due to the truncation of N- and C-terminal regions in the structures. Hence, they will not be discussed further.

To uniquely define the position of residues in any AQP structure, we have earlier proposed a structure-based generic numbering scheme (1) for comparing residue positions in diverse AQP sequences. In this scheme, the most conserved position in each of the six TM helical segments and the two half-helices are identified from a large number of MIP sequences. This conserved residue within a TM helix is given the number 50, and all other positions in the same TM helix are relative to this position. Thus, the 3.47 and 5.52 residue positions refer to the three and two residues positions preceding and succeeding the most conserved residues of the third and fifth TM helices, respectively. The most conserved residues in each of the TM helix and the half-helices are shown in Figure 1, and this generic numbering scheme will be used hereafter. A similar generic numbering scheme has been used for G-Protein Coupled Receptors and transporters (57, 66). Very recently, a generic number scheme is proposed for AQPs also along the similar lines (67).

For each SNP that resulted in missense mutation, we examined where exactly they occurred in the structure. For this purpose, we divided the AQP structure into six different regions, namely, (i) channel-facing, (ii) helix–helix interface, (iii) monomer–monomer interface, (iv) lipid-facing, (v) exposed to the cytoplasm or (vi) exposed to the extracellular environment.

The NPA motifs and the residues forming the Ar/R SF are functionally important regions. Apart from these two regions, the classification of six regions with distinct structural features is based on the following criteria. To find out the membrane boundary in the AQP TM segments, we used the Orientation of Proteins in Membranes (https://opm.phar.umich.edu/) and Positioning of Proteins in Membranes (https://opm.phar.umich.edu/ppm_server) servers (68). Residues are classified as channel-facing if both backbone and side-chain atoms face the channel interior. If the backbone of a residue in a TM helix is present within 4 Å of another TM helix, then this residue is considered as a residue at the helix–helix interface. If a residue of one monomer is present within 4 Å of another monomer, then this residue is considered to be present at the monomer–monomer interface. If a residue is present inside the TM region and not at the monomer–monomer interface or any of the other regions mentioned earlier, then it is considered as lipid-facing. If the residues are present outside the membrane region and toward the extracellular environment (or the cytoplasm), then these residues are considered to be exposed to the extracellular (or cytoplasmic) environment. The nature of residue changes in functionally important positions and other structural regions are discussed in the following subsections.

### NPA motifs

The NPA motif is highly conserved in AQPs in LB and LE half-helices and has been shown to be functionally important (9, 10). It is one of the two narrowest regions in AQP channels. Hence, any change in this motif is likely to have repercussions on the transport properties of AQPs. Missense mutations due to SNPs have been observed in both NPA motifs. There are 33 and 23 instances in which substitutions occur in NPA motifs found in LB and LE half-helices, respectively, and they are summarized in Table 2. Simulation and experimental studies have shown that the side chain of Asn in NPA motifs prevents the transport of protons and cations in AQP channels (69–71). Asn in the NPA motifs is completely invariant in all human AQP homologs. Missense variation due to SNPs results in the substitution of Asn in the NPA motifs (LB.50 and LE.50 positions) to Thr, Ser, Asp, Ile and Lys. The replacement of Asn by residues such as Lys and Ile is most likely to affect the function of human AQPs AQP1, AQP9, AQP10 and AQP11 (Table 2).

**Table 2.** Missense SNP substitutions in functionally important regions of human AQP homologs

Functionally important regions	SNPs observed^a^
LB-NPA motif	Asn^LB.50^ → Thr (AQP2, AQP5, AQP6, AQP11), Ser (AQP2, AQP8, AQP9, AQP11), Asp (AQP9), Ile (AQP9, AQP11), Lys (AQP10) Pro^LB.51^ → Thr (AQP0, AQP12), Gln (AQP1, AQP11), Leu (AQP1, AQP10, AQP11), Ala (AQP1), Ser (AQP1, AQP3), His (AQP5), Arg (AQP12) Ala^LB.50^ → Thr (AQP7) Ala^LB.52^ → Val (AQP3, AQP8, AQP10), Ser (AQP5), Thr (AQP6), Asp (AQP6), Gly (AQP7) Thr^LB.52^ → Ala (AQP12)
LE-NPA motif	Asn^LE.50^ → Thr (AQP0, AQP7), Lys (AQP1, AQP9), Ser (AQP2, AQP7, AQP10) Pro^LE.51^ → Ser (AQP0, AQP4), Ala (AQP2, AQP5, AQP10), Arg (AQP5, AQP9), Leu (AQP5, AQP7) Ala^LE.52^ → Thr (AQP4, AQP8, AQP9), Val (AQP5, AQP10), Asp (AQP8) Ser^LE.52^ → Phe (AQP7)
Ar/R SF	Phe^2.49^ → Cys (AQP0), Leu (AQP1), Ile (AQP2) His^2.49^ → Gln (AQP8), Tyr (AQP8) Tyr^2.49^ → Cys (AQP11) Ala^LE.47^ → Thr (AQP0, AQP4) Cys^LE.47^ → Phe (AQP1), Trp (AQP2, AQP5, AQP6, AQP9), Tyr (AQP2) Tyr^LE.47^ → Cys (AQP3) Ile^LE.47^ → Thr (AQP10) Arg^LE.53^ → Leu (AQP0, AQP4, AQP7), His (AQP0, AQP2, AQP5, AQP6, AQP8), Cys (AQP0, AQP2, AQP5, AQP6, AQP8), Gly (AQP1), Trp (AQP1, AQP3, AQP7, AQP10), Gln (AQP1, AQP3, AQP4, AQP7, AQP9, AQP10), Ser (AQP6), Pro (AQP8, AQP9) His^5.57^ → Gln (AQP2), Arg (AQP4, AQP5), Tyr (AQP4) Gly^5.57^ → Arg (AQP7, AQP10), Ala (AQP7) Ile^5.57^ → Met (AQP8) Ala^5.57^ → Val (AQP9), Thr (AQP12) Val^5.57^ → Ile (AQP11)

a The generic number for each residue and the substituted amino acid residue due to missense SNPs is shown. The human AQPs in which this substitution occurs are shown within brackets.

SNPs that give rise to missense variation lead to the substitution of proline residues (LB.51 and LE.51 positions) in NPA motifs by Thr, Gln, Leu, Ala, Ser, His and Arg. Proline in NPA motifs plays a structural role as the N-cap residue for the half-helices found in LB and LE loops. Hence, the substitution of proline by any other residue is likely to affect the helix stability. The proline residue in the NPA motif of LB is replaced by Ala in wild-type AQP7. Ala at LB.51 in wild-type AQP7 is substituted by Thr due to missense SNP. Since Thr has a hydroxyl group in its side chain, it will be interesting to see how this will affect the transport properties in AQP7.

The highly conserved Ala in the LB.52 position of NPA motifs is substituted by Cys and Thr in wild-type AQP11 and AQP12, respectively. Similarly, Ala in the LE.52 position is replaced by Ser in wild-type AQP7. All other human AQP homologs have Ala in LB.52 and LE.52 positions. Alanine’s substitution in NPA motifs to Thr, Ser, Asp, Val and Gly alters either the chemical nature of one of the constrictions in the channel or a bulkier residue like Val introduces further constriction in the channel. In the case of AQP12, the wild-type Thr at Position LB.52 is replaced by Ala, whereas Ser at LE.52 in AQP7 is substituted by the bulky aromatic residue Phe. The substitutions at LB.52 and LE.52, respectively, in AQP12 and AQP7 are likely to affect the transport properties. Hence, we can easily speculate that missense mutations in any of the positions corresponding to the conserved NPA motifs will have serious consequences that can compromise the transport properties of AQPs.

### Aromatic/arginine selectivity filter

The Ar/R SF forms the most important constriction in AQP channels. The Ar/R SF is formed by four residues, one each from TM2 and TM5 helices and two residues from the LE loop. In the generic numbering scheme, the positions of these residues are 2.49, 5.57, LE.47 and LE.53 (1). Mutation and computational studies suggest that the replacement of residues belonging to the Ar/R SF results in different transport properties in AQP channels (11, 12, 72, 73). As the name suggests, in 11 out of 13 human AQP homologs, Arg is present in the LE.53 position. In AQP11 and AQP12, Arg is replaced by Leu. With the exception of AQP10 and AQP12, aromatic residues Phe, His or Tyr are present in Position 2.49. In the other two positions, namely 5.57 and LE.47, many types of residues are found. SNPs result in substitutions in all four positions that form the Ar/R SF and are summarized in Table 2. Missense variations due to SNPs are found in the LE.53 position in which the functionally important Arg is replaced by residues that have completely different chemical and physical properties (Leu, His, Cys, Gly, Trp, Gln, Ser and Pro), implying that the selectivity and transport will be certainly compromised. As far as the 2.49 position is concerned, the aromatic residues Phe and Tyr are substituted by Cys (AQP0 and AQP11) or hydrophobic residues (AQP1 and AQP2). In the case of AQP8, the missense mutations due to SNPs result in the substitution of His^2.49^ by Gln or Tyr. The LE.47 position is occupied by Cys in five human homologs, namely, AQP1, AQP2, AQP5, AQP6 and AQP9. SNPs at this position result in the substitution of bulky aromatic residues. Such replacement is most likely to restrict the size of the substrate that will be transported through these AQP homologs. At Position 5.57, six human AQP homologs have His, three have Gly and the remaining have hydrophobic residues. In three human AQPs, His^5.57^ is replaced by Gln (AQP2), Arg (AQP4 and AQP5) and Tyr (AQP4), indicating that these changes could affect the function. The missense mutations due to SNPs give rise to the substitution of Gly^5.57^ in AQP7 and AQP10 to Arg, and this will surely impact the type of substrates that will be transported along these channels.

### Channel-facing residues

There are 287 missense mutations involving residues that can be characterized as channel-facing. Overall, 9% of all the SNPs occur in the channel-facing positions, indicating that drastic changes in these positions are likely to affect the transport properties of AQPs. In addition to NPA motifs and residues that are part of the Ar/R SF, other positions from which side chains of residues directly point to the channel axis are likely to interact with the permeating substrates and could certainly influence the transport across the human AQP channels. Among all human AQPs, AQP2 and AQP7 have the maximum number of 33 and 30 instances, respectively, in which the channel-facing wild-type residues are replaced due to SNPs. Substitutions of channel-facing residues that can be considered as non-conservative are listed in Table 3. Some of the notable substitutions include those at Positions 1.53, 3.42, 4.65 and 6.62 in which charged residues Arg/Lys are replaced by aromatic or small residues. At Position 4.66, the negatively charged Asp in AQP6 is mutated to positively charged Arg, while the positively charged Lys^1.69^ is substituted by negatively charged Glu in AQP1. Similarly, Gly at Positions 1.61, 5.61 and 5.65 are substituted by bulkier or charged residues. As these substitutions at positions facing the channel drastically change the chemical and/or physical properties of the residues, we can speculate that they could alter the selectivity and transport of substrates compared to the wild-type proteins that will result in different phenotypes.

**Table 3.** Missense mutations due to SNPs in channel-facing residues

Channel-facing residues^a^	Missense mutations due to SNPs^b^
TM1	Arg^1.53^ → Trp (AQP12); Phe^1.57^ → Ser (AQP5) Leu^1.57^ → Ser (AQP9), Pro (AQP10) Gly^1.61^ → Arg (AQP2, AQP8), Val (AQP7, AQP9), Glu (AQP9) Thr^1.61^ → Ile (AQP11); Ala^1.65^ → Asp (AQP1); Val^1.65^ → Gly (AQP9) Gln^1.65^ → Arg (AQP11); Tyr^1.65^ → Asn (AQP12), His (AQP12) Met^1.68^ → Thr (AQP7), Lys (AQP7); Lys^1.69^ → Glu (AQP1) Ile^1.69^ → Thr (AQP9)
TM2	Ile^2.45^ → Thr (AQP2, AQP6), Ser (AQP5); Pro^2.45^ → Leu (AQP8) Tyr^2.49^ → Cys (AQP11); Leu^2.53^ → Pro (AQP0, AQP11) Ile^2.53^ → Thr (AQP1, AQP4), Ser (AQP4, AQP5); Thr^2.53^ → Ile (AQP6) Val^2.57^ → Gly (AQP7); Ile^2.57^ → Ser (AQP9), Asn (AQP10) Trp^2.61^ → Arg (AQP6); Gly^2.61^ → Val (AQP7), Asp (AQP7), Arg (AQP8) Ser^2.64^ → Arg (AQP2); Gly^2.65^ → Arg (AQP2)
LB	Val ^HB.53^ → Asp (AQP0); Cys ^HB.57^ → Tyr (AQP2); Met ^HB.57^ → Thr (AQP10)
TM3	Arg^3.42^ → Cys (AQP0, AQP6), Gly (AQP0), Trp(AQP5) Lys^3.42^ → Asn (AQP3, AQP4), Thr (AQP9); Thr^3.42^ → Met (AQP11)
TM4	Leu^4.57^ → Pro (AQP6); Val^4.61^ → Asp (AQP9); Leu^4.61^ → Pro (AQP12) Thr^4.65^ → Ile (AQP2), Met (AQP7); Arg^4.65^ → Trp (AQP12), Gln (AQP12); Asp^4.66^ → Gly (AQP6)
TM5	Ile^5.45^ → Thr (AQP4), Lys (AQP4) Ile^5.49^ → Thr (AQP2, AQP4, AQP6, AQP7, AQP9), Asn (AQP9), Ser (AQP9) Val^5.53^ → Asp (AQP8, AQP12); Gly^5.57^ → Arg (AQP10) Met^5.61^ → Thr (AQP0); Ile^5.61^ → Thr (AQP1) Gly^5.61^ → Val (AQP7, AQP11), Arg (AQP12) Gly^5.65^ → Arg (AQP2), Asp (AQP4), Val (AQP5)
TM6	Phe^6.62^ → Ser (AQP5); Arg^6.62^ → Ser (AQP8)

a The TM segments and the half-helix regions in the hour-glass helical fold are given.

b The generic number of the channel-facing residue and its substitution due to missense SNPs are shown here. The human AQPs in which this substitution occurs are shown within brackets.

### Helix–helix interface

We have previously shown that the residues belonging to the SWP group are close to 95% group conserved at the helix–helix interface of many membrane proteins including AQPs, formate/nitrite transporters, Sugars Will Eventually be Exported Transporters (55–57). The presence of such residues at the helix–helix interface helps to bring the TM helices close together for an optimal helix–helix packing interaction. As mentioned earlier, we define a residue at the helix–helix interface if the backbone of the residue of one helix is within 4 Ǻ of another helix. The maximum number of >850 SNPs occurs at the helix–helix interface. When we examined the human AQP SNPs that occur at the helix–helix interface, half of all SNPs in human AQP homologs involve SWP residues. Among them, 174 involve missense mutations that result in a change of one SWP to another SWP residue. In the remaining 254 cases, SWP residues at the helix–helix interface are substituted by bulkier hydrophobic or aromatic residues, indicating that the helix-bundle geometry is likely to be disrupted with such replacement. This may lead to the overall destabilization of the hour-glass fold typically found in AQP structures. Similarly in ∼50 SNPs, AH residues are substituted by SWP residues that may have an effect on the helix packing. The introduction of a charged residue requires another charged/polar residue within the TM helical domain so that the two residues can interact among themselves. Otherwise, their presence in a hydrophobic environment becomes unfavorable (74). Hence, the substitution of any hydrophobic residue by a charged residue will be energetically not favorable within the TM region. The same is true when a charged residue is substituted by a hydrophobic residue in the middle of the hydrophobic environment. There are close to 164 entries in which charged residues are introduced at the helix–helix interface. However, we found <15 examples in which the missense mutation of charged residue leads to hydrophobic/aromatic/SWP residues. The introduction of a charged residue in the TM region or replacement of a charged residue by hydrophobic/aromatic residues will destabilize a membrane helix-bundle protein.

### Missense mutations in other positions

We also analyzed positions that occur at the monomer–monomer interface and lipid-facing positions. There are 371 entries that can be defined to fall at the interface of two monomers in the tetrameric arrangement of AQPs. It has been shown that the function of AQP monomers can be influenced by their interaction with the neighboring monomers in the tetramer assembly (75, 76). Hence, missense variations due to SNPs at the monomer–monomer interface could potentially impact the transport properties of human AQP homologs. Another 294 SNPs are found in positions that are lipid-exposed. The majority of them involve AH residues. These are mostly substituted by another hydrophobic residue from the same group. These substitutions are likely to have little effect on the structure and function of AQPs.

Helices of AQP hour-glass-shaped helix-bundle extend beyond the lipid head–group region, and thus these are exposed to the cytoplasmic or extracellular side. We have looked at the positions of these exposed helical regions and found 122 and 85 cases, respectively, in which SNPs occur in these regions. The majority of these SNPs result in missense mutations involving SWP or charged residues. We have identified 718 SNPs that are found in the loop regions connecting the six TM helices. This is the second largest category after those found at the helix–helix interface. Not surprisingly, >500 of these SNPs involve residues that are classified as SWP, charged or NP. Hence, we speculate that these substitutions will not have any major consequence on the structure and/or function of the human MIPs.

SNPs were found at positions at the N- or C-terminal regions that are within the structurally defined regions. We found >40 and 90 examples in N- or C-terminal tails, respectively, in which missense mutations due to SNPs occur. Compared to functionally recognized regions such as NPA motifs and Ar/R SF residues, structural and/or functional consequences of missense variations due to SNPs will be difficult to speculate in N- and C-terminal regions. If the residues are known to undergo phosphorylation or other post-translational modifications (PTMs), then substitutions in these positions will have a significant effect on the function or regulation of these channel proteins. However, residues undergoing PTMs have to be clearly established.

## Discussion

Several studies have investigated the association between human AQP SNPs and health complications or diseases in specific populations (17–43). This includes more prevalent problems such as Type 2 diabetes, hypertension and obesity. Human AQP SNPs have been implicated in other health risks such as acute body fluid loss, liver cirrhosis, periodontitis, temporomandibular joint disorders, cerebral edema, sleep-related disorders, sudden infant death syndrome, neuromyelitis optica, vascular depression, schizophrenia, chronic obstructive pulmonary disease, pathogenesis of caries, adverse kidney events, polycystic ovary syndrome and femoral neck bone mineral density. Many of these missense SNPs that are found to be associated with diseases or health complications are not available in databases like OMIM and Humsavar, which are part of the UniProt/Swiss-Prot protein knowledgebase (https://www.uniprot.org).

Calvanese *et al.* considered 34 single amino acid polymorphisms found in human AQPs that are associated with genetic disorders and investigated the possible relationship between the structural defects and experimental phenotypes for 17 mutations (77). The database MutHTP (78) has compiled details of disease-associated and neutral mutations from ∼5000 human TM proteins. As per the ‘Statistics’ page of this website, the total number of distinct disease mutations is 183 395, and the number of neutral mutations is 17 827. The mutation data were collected from different mutation databases such as Humsavar, SwissVar (79), 1000 Genomes (80), COSMIC (81) and ClinVar (54). This implies that on average, there are ∼40 disease mutations per TM protein. We realize that some proteins may have higher disease mutations, and some may have a negligible number of disease mutations. Recently, a study by Iqbal *et al.* (60) identified 3D features associated with pathogenic (disease-associated) and population (benign) missense variants from 1330 disease-associated genes using 14 270 experimentally determined structures. The mutation data for this study was compiled from OMIM (53), Human Gene Mutation Database (82), ClinVar (54), Exome Aggregation Consortium (83) and Genome Aggregation Database (84). The 3D features include the mutated amino acids’ physicochemical properties, structural context and functional features. Among the different functional classes they analyzed, four AQPs (AQP1, AQP2, AQP4 and AQP5) have been included under the ‘Transporter’ functional class. In all four AQP homologs, only 58 pathogenic missense variants are available, while population (neutral) missense variants are 542 in total out of which 447 have been mapped onto the 3D structure. Hence, the number of pathogenic mutants available in the mutation databases for the AQP family is in general very less. However, there are many reports associating SNPs with disease conditions as detailed in the Introduction section. In the current study, we have found >20 examples in the OMIM database in which AQP SNPs result in diseases. Most of these missense SNPs occur in the helix–helix interface or they are facing the channel interior, and one can safely assume that the substitutions due to SNPs could possibly interfere with the structure and/or function. Other possibilities include improper folding and targeting. The study by Igbal *et al.* showed that the 3D-mutational hotspots can be different across different protein structural and functional classes (60). In this regard, the current study is very important as it has compiled the missense variants and classified functional features specific to AQP family members. As mentioned above, at present the number of recognized pathogenic variants for the AQP family is very less as evident from other studies. When more pathogenic variants are recognized, the current data with structural and functional classifications can be applied to come up with the prediction for human AQP missense substitutions.

In this study, a systematic search in the dbSNP yielded ∼2800 missense SNPs in all human AQP homologs. Hence, it is important to analyze the nature of the residue substitutions that can help to infer where the substitutions in the structure take place and what kind of substitutions are occurring. We are fully aware that not all SNPs will result in pathogenic conditions. However, it is important to know what kind of substitutions occur due to missense SNPs and whether or not these substitutions are likely to affect the structure and/or function of human AQPs. We analyzed all the missense SNPs of human AQPs to understand the pattern of substitutions. We divided the naturally occurring amino acids into six groups, and this classification is based on both chemical and physical properties. This analysis helped us to find out some of the unusual and non-conservative substitutions which included small to big, hydrophobic to charged and negatively charged to positively charged, and vice versa. Some substitutions are never observed. For example, we did not find even a single case in which aromatic amino acids are substituted by proline, and vice versa. We have also compared the pattern of substitutions found in our study and the data available in the MutHTP database (78). A comparison between data obtained from mutation databases and the current study using SNP missense substitution data reveals that there are some notable differences in the pattern of amino acid substitutions. For example, in the present study, we have not found even a single example in which NP (Gln and Asn) residues are replaced by aromatic residues (Tyr, Trp and Phe). However, in MutHTP, many examples have been found in which Asn is replaced by Tyr in disease mutations, and a few cases of Asn → Tyr mutation were found in neutral mutations. Similarly, Pro to Aromatic substitutions and vice versa were not found in the present study. In MutHTP, there are some examples in which Phe is substituted by Pro in disease mutations.

Next, we wanted to find out where exactly these substitutions occur with respect to the structure. We found many examples of non-conservative substitutions in the functionally important NPA motif or Ar/R SF. Similarly, substitutions that are likely to disrupt the TM helix packing occur at the helix–helix interface. Building the dbAQP-SNP database and making it publicly available is the first step where researchers can use this resource to look at specific missense SNPs, the type of substitutions and the structural region in which these substitutions occur. Using the Advanced Search options, one can look at all the missense SNPs that occur in specific structural regions. One can also choose a particular human AQP homolog and focus on all missense SNPs to understand whether these SNPs are likely to result in pathogenic conditions. We hope that this resource will help to further advance our knowledge in associating SNPs in human AQPs with the structural and functional defects that may be pathogenic.

## Supplementary Material

baad012_Supp

## References

[1] Verma R.K. , Gupta A.B. and Sankararamakrishnan R. (2015) Major intrinsic protein superfamily: channels with unique structural features and diverse selectivity. *Methods Enzymol.*, 557, 485–520. 25950979 10.1016/bs.mie.2014.12.006

[2] Abascal F. , Irisarri I. and Zardoya R. (2014) Diversity and evolution of membrane intrinsic proteins. *Biochim. Biophys. Acta*, 1840, 1468–1481. 24355433 10.1016/j.bbagen.2013.12.001

[3] Finn R.N. and Cerda J. (2015) Evolution and functional diversity of aquaporins. *Biol. Bull.*, 229, 6–23. 26338866 10.1086/BBLv229n1p6

[4] Park W. , Scheffler B.E., Bauer P.J. et al. (2010) Identification of the family of aquaporin genes and their expression in upland cotton (*Gossypium hirsutum* L). *BMC Plant Biol.*, 10, Art. No. 142. 10.1186/1471-2229-10-142 PMC3095289 20626869

[5] Gupta A.B. and Sankararamakrishnan R. (2009) Genome-wide analysis of major intrinsic proteins in the tree plant *Populus trichocarpa*: characterization of XIP subfamily of aquaporins from evolutionary perspective. *BMC Plant Biol.*, 9, Art. No. 134. 10.1186/1471-2229-9-134 PMC2789079 19930558

[6] Verma R.K. , Prabh N.D. and Sankararamakrishnan R. (2014) New subfamilies of major intrinsic proteins in fungi suggest novel transport properties in fungal channels: implications for the host-fungal interactions. *BMC Evol. Biol.*, 14, Art. No. 173. 10.1186/s12862-014-0173-4 PMC4236510 25112373

[7] Azad A.K. , Raihan T., Ahmed J. et al. (2021) Human aquaporins: functional diversity and potential roles in infectious and non-infectious diseases. *Front. Genet.*, 12, Art. No. 654865. 10.3389/fgene.2021.654865 PMC8007926 33796134

[8] Ishibashi K. , Tanaka Y. and Morishita Y. (2021) The role of mammalian superaquaporins inside the cell: an update. *Biochim. Biophys. Acta*, 1863, Article No. 183617. 10.1016/j.bbamem.2021.183617 33811846

[9] Eriksson U.K. , Fischer G., Friemann R. et al. (2013) Subangstrom resolution X-ray structure details aquaporin-water interactions. *Science*, 340, 1346–1349. 23766328 10.1126/science.1234306 PMC4066176

[10] de Groot B.L. and Grubmuller H. (2001) Water permeation across biological membranes: mechanism and dynamics of aquaporin-1 and GlpF. *Science*, 294, 2353–2357. 11743202 10.1126/science.1066115

[11] Beitz E. , Wu B.H., Holm L.M. et al. (2006) Point mutations in the aromatic/arginine region in aquaporin 1 allow passage of urea, glycerol, ammonia, and protons. *Proc. Natl. Acad. Sci. USA*, 103, 269–274. 16407156 10.1073/pnas.0507225103 PMC1326162

[12] Mitani-Ueno N. , Yamaji N., Zhao F.J. et al. (2011) The aromatic/arginine selectivity filter of NIP aquaporins plays a critical role in substrate selectivity for silicon, boron, and arsenic. *J. Exp. Botany*, 62, 4391–4398. 21586431 10.1093/jxb/err158 PMC3153687

[13] Chen G. , Zhang Z.L., Shang R.S. et al. (2018) In vitro expression and functional characterization of NPA motifs in aquaporins of *Nosena bombycis*. *Parasitol. Res.*, 117, 3473–3479. 30105406 10.1007/s00436-018-6044-y

[14] Verma R.K. , Prabh N.D. and Sankararamakrishnan R. (2015) Intra-helical salt-bridge and helix destabilizing residues within the same helical turn: role of functionally important loop E half-helix in channel regulation of major intrinsic proteins. *Biochim. Biophys. Acta*, 1848, 1436–1449. 25797519 10.1016/j.bbamem.2015.03.013

[15] Jain A. , Verma R.K. and Sankararamakrishnan R. (2019) Presence of intra-helical salt-bridge in loop E half-helix can influence the transport properties of AQP1 and GlpF channels: molecular dynamics simulations of in silico mutants. *J. Memb. Biol.*, 252, 17–29. 10.1007/s00232-018-0054-7 30470864

[16] Agre P. , King L.S., Yasui M. et al. (2002) Aquaporin water channels—from atomic structure to clinical medicine. *J. Physiol. London*, 542, 3–16. 12096044 10.1113/jphysiol.2002.020818 PMC2290382

[17] Sorani M.D. , Manley G.T. and Giacomini K.M. (2008) Genetic variation in human aquaporins and effects on phenotypes of water homeostasis. *Hum. Mutat.*, 29, 1108–1117. 18470935 10.1002/humu.20762

[18] Elliott L. , Ashley-Koch A.E., De Castro L. et al. (2007) Genetic polymorphisms associated with priapism in sickle cell disease. *Br. J. Haematol.*, 137, 262–267. 17408468 10.1111/j.1365-2141.2007.06560.x

[19] Rivera M.A. , Martinez J.L., Carrion A. et al. (2011) AQP-1 association with body fluid loss in 10-km runners. *Int. J. Sports Med.*, 32, 229–233. 21271497 10.1055/s-0030-1268489

[20] Fabrega E. , Berja A., Garcia-Unzueta M.T. et al. (2011) Influence of aquaporin-1 gene polymorphism on water retension in liver cirrhosis. *Scand. J. Gastroenterol.*, 46, 1267–1274. 21793635 10.3109/00365521.2011.603161

[21] Wang Y. , Yin J.-Y., Li X.-P. et al. (2014) The association of transporter genes polymorphisms and lung cancer chemotherapy response. *PLoS One*, 9, Art. No. e91967. 10.1371/journal.pone.0091967 PMC3958404 24643204

[22] Bezamat M. , Cunha E.J., Modesto A.M. et al. (2020) Aquaporin locus (12q13.12) might contribute to susceptibility of temporomandibular joint disorder associated with periodontitis. *PLoS One*, 15, Art. No. e0229245. 10.1371/journal.pone.0229245 PMC7055872 32130259

[23] Kang S. , Chae Y.S., Lee S.J. et al. (2015) Aquaporin 3 expression predicts survival in patients with HER2-positive early breast cancer. *Anticancer Res.*, 35, 2775–2782. 25964557

[24] Sorani M.D. , Zador Z., Hurowitz E. et al. (2008) Novel variants in human Aquaporin-4 reduce cellular water permeability. *Hum. Mol. Genet.*, 17, 2379–2389. 18511455 10.1093/hmg/ddn138 PMC2733814

[25] Rainey-Smith S.R. , Mazzucchelli G.N., Villemagne V.L. et al. (2018) Genetic variation in Aquaporin-4 moderates the relationship between sleep and brain Aβ-amyloid burden. *Transl. Psychiatry*, 8, Art. No. 47. 10.1038/s41398-018-0094-x PMC5865132 29479071

[26] Larsen S.M.U. , Landolt H.-P., Berger W. et al. (2020) Haplotype of the astrocytic water channel AQP4 is associated with slow wave energy regulation in human NREM sleep. *PLoS Biol.*, 18, Art. No. e3000623. 10.1371/journal.pbio.3000623 PMC7199924 32369477

[27] Wu Y.-F. , Sytwu H.-K. and Lung F.-W. (2018) Human aquaporin 4 gene polymorphisms and haplotypes are associated with serum 100B level and negative symptoms of schizopherenia in a southern Chinese Han population. *Front. Psychiatry*, 9, Art. No. 657. 10.3389/fpsyt.2018.00657 PMC6297372 30618856

[28] Studer J. , Bartsch C. and Haas C. (2014) Aquaporin-4 polymorphisms and brain/body weight ratio in sudden infant death syndrome (SIDS). *Pediatr. Res.*, 76, 41–45. 24727946 10.1038/pr.2014.59

[29] Matiello M. , Schaefer-Klein J.L., Hebrink D.D. et al. (2011) Genetic analysis of aquaporin-4 in neuromyelitis optica. *Neurology*, 77, 1149–1155. 21900637 10.1212/WNL.0b013e31822f045b

[30] Westermair A.L. , Munz M., Schaich A. et al. (2018) Association of genetic variation at AQP4 locus with vascular depression. *Biomolecules*, 8, Art. No. 164. 10.3390/biom8040164 PMC6316852 30563176

[31] Wu Y.-F. , Sytwu H.-K. and Lung F.-W. (2020) Polymorphisms in the human Aquaporin 4 gene are associated with schizophrenia in the southern Chinese Han population: a case–control study. *Front. Psychiatry*, 11, Art. No. 596. 10.3389/fpsyt.2020.00596 PMC7333661 32676041

[32] Dardiotis E. , Siokas V., Marogianni C. et al. (2019) AQP4 tag SNPs in patients with intracerebral hemorrhage in Greek and Polish population. *Neurosci. Lett.*, 696, 156–161. 30578930 10.1016/j.neulet.2018.12.025

[33] Ning Y. , Ying B., Han S. et al. (2008) Polymorphisms of aquaporin5 gene in chronic obstructive pulmonary disease in a Chinese population. *Swiss Med. Wkly.*, 138, 573–578. 18853286 10.4414/smw.2008.12240

[34] Hansel N.N. , Sidhaye V., Rafaels N.M. et al. (2010) Aquaporin 5 polymorphisms and rate of lung function decline in chronic obstructive pulmonary disease. *PLoS One*, 5, Art. No. e14226. 10.1371/journal.pone.0014226 PMC2997058 21151978

[35] Wang X. , Willing M.C., Marazita M.L. et al. (2012) Genetic and environmental factors associated with dental caries in children: the Iowa fluoride study. *Caries Res.*, 46, 177–184. 22508493 10.1159/000337282 PMC3580152

[36] Vieira A.R. , Bayram M., Seymen F. et al. (2017) In vitro acid-mediated initial dental enamel loss is associated with genetic variants previously linked to caries experience. *Front. Physiol.*, 8, Art. No. 104. 10.3389/fphys.2017.00104 PMC5319969 28275354

[37] Bergmann L. , Nowak H., Siffert W. et al. (2020) Major adverse kidney events are associated with the aquaporin 5-1364A/C promoter polymorphism in sepsis: a prospective validation study. *Cells*, 9, Art. No. 904. 10.3390/cells9040904 PMC7226758 32272738

[38] Kasimir-Bauer S. , Heubner M., Otterbach F. et al. (2009) Prognostic relevance of the AQP5 −1364C>A polymorphism in primary breast cancer. *Mol. Med. Rep.*, 2, 645–650. 21475880 10.3892/mmr_00000151

[39] Prudente S. , Flex E., Morini E. et al. (2007) A functional variant of the adipocyte glycerol channel aquaporin 7 gene is associated with obesity and related metabolic abnormalities. *Diabetes*, 56, 1468–1474. 17351148 10.2337/db06-1389

[40] Wang Y. , Chen G., Tu Q. et al. (2018) Associations between aquaglyceroporin gene polymorphisms and risk of type 2 diabetes mellitus. *Biomed Res. Intl.*, 2018, Art. No. 8167538. 10.1155/2018/8167538 PMC6288565 30598999

[41] Tu Q. , Yan L., Wang C. et al. (2020) Associations between aquaglyceroporin gene polymorphisms and risk of stroke among patients with hypertension. *BioMed. Res. Int.*, 2020, Art. No. 9358290. 10.1155/2020/9358290 PMC7136773 32309443

[42] Li Y. , Liu H., Zhao H. et al. (2013) Association of AQP8 in women with PCOS. *Reprod. BioMed. Online*, 27, 419–422. 23953589 10.1016/j.rbmo.2013.07.001

[43] Chanprasertyothin S. , Saetung S., Rajatanavin R. et al. (2010) Genetic variant in the aquaporin 9 gene is associated with bone mineral density in postmenopausal women. *Endocrine*, 38, 83–86. 20960106 10.1007/s12020-010-9353-1

[44] Stajnko A. , Slejkovec Z., Mazej D. et al. (2019) Arsenic metabolites; selenium; and AS3MT, MTHFR, AQP4, AQP9, SELENOP, INMT, and MT2A polymorphisms in Croatian-Slovenian population from PHIME-CROME study. *Environ. Res.*, 170, 301–319. 30612060 10.1016/j.envres.2018.11.045

[45] Sherry S.T. , Ward M.-H., Kholodov M. et al. (2001) dbSNP: the NCBI database of genetic variation. *Nucleic Acids Res.*, 29, 308–311. 11125122 10.1093/nar/29.1.308 PMC29783

[46] O’Leary N.A. , Wright M.W., Brister J.R. et al. (2016) Reference sequence (RefSeq) database at NCBI: current status, taxonomic expansion, and functional annotation. *Nucleic Acids Res.*, 44, D733–D745. 26553804 10.1093/nar/gkv1189 PMC4702849

[47] Rose A.S. and Hildebrand P.W. (2015) NGL viewer: a web application for molecular visualization. *Nucleic Acids Res.*, 43, W576–W579. 25925569 10.1093/nar/gkv402 PMC4489237

[48] Pettersen E.F. , Goddard T.D., Huang C.C. et al. (2004) UCSF Chimera—a visualization system for exploratory research and analysis. *J. Comput. Chem.*, 25, 1605–1612. 15264254 10.1002/jcc.20084

[49] Shapovalov M.V. and Dunbrack R.L. Jr. (2011) A smoothed backbone-dependent rotamer library for proteins derived from adaptive kernel density estimates and regressions. *Structure*, 19, 844–858. 21645855 10.1016/j.str.2011.03.019 PMC3118414

[50] Goji K. , Kuwahara M., Gu Y. et al. (1998) Novel mutations in aquaporin-2 gene in female siblings with nephrogenic diabetes insipidus: evidence of disrupted water channel function. *J. Clin. Endocrinol. Metab.*, 83, 3205–3209. 9745427 10.1210/jcem.83.9.5074

[51] Blaydon D.C. , Lind L.K., Plagnol V. et al. (2013) Mutations in AQP5, encoding a water-channel protein, cause autosomal-dominant diffuse nonepidermolytic palmoplantar keratoderma. *Am. J. Hum. Genet.*, 93, 330–335. 23830519 10.1016/j.ajhg.2013.06.008 PMC3738836

[52] Mulders S.M. , Bichet D.G., Rijss J.P. et al. (1998) An aquaporin-2 water channel mutant which causes autosomal dominant nephrogenic diabetes insipidus is retained in the Golgi complex. *J. Clin. Invest.*, 102, 57–66. 9649557 10.1172/JCI2605 PMC509065

[53] Hamosh A. , Scott A.F., Amberger J.S. et al. (2005) Online Mendelian Inheritance in Man, a knowledgebase of human genes and genetic disorders. *Nucleic Acids Res.*, 33, D514–D517. 15608251 10.1093/nar/gki033 PMC539987

[54] Landrum M.J. , Lee J.M., Riley G.R. et al. (2014) ClinVar: public archive of relationships among sequence variation and human phenotype. *Nucleic Acids Res.*, 42, D980–D985. 24234437 10.1093/nar/gkt1113 PMC3965032

[55] Bansal A. and Sankararamakrishnan R. (2007) Homology modeling of major intrinsic proteins in rice, maize and *Arabidopsis*: comparative analysis of transmembrane helix association and aromatic/arginine selectivity filters. *BMC Struct. Biol.*, 7, Art. No. 27. 10.1186/1472-6807-7-27 PMC1866351 17445256

[56] Mukherjee M. , Vajpai M. and Sankararamakrishnan R. (2017) Anion-selective formate/nitrite transporters: taxonomic distribution, phylogenetic analysis and subfamily-specific conservation pattern in prokaryotes. *BMC Genom.*, 18, Art. No. 560. 10.1186/s12864-017-3947-4 PMC5525234 28738779

[57] Gupta A. and Sankararamakrishnan R. (2018) dbSWEET: an integrated resource for SWEET superfamily to understand, analyze and predict the function of sugar transporters in prokaryotes and eukaryotes. *J. Mol. Biol.*, 430, 2203–2211. 29665371 10.1016/j.jmb.2018.04.013

[58] Sankararamakrishnan R. and Vishveshwara S. (1992) Geometry of proline-containing alpha-helices. *Int. J. Pept. Protein Res.*, 39, 356–363. 1428525 10.1111/j.1399-3011.1992.tb01595.x

[59] Ghosh T.S. , Chaitanya S.K. and Sankararamakrishnan R. (2009) End-to-end and end-to-middle inter-helical interactions: new classes of interacting helix pairs in protein structures. *Acta Crystallogr. D*, 65, 1032–1041. 19770500 10.1107/S0907444909027012 PMC2756166

[60] Iqbal S. , Perez-Palma E., Jespersen J.B. et al. (2020) Comprehensive characterization of amino acid positions in protein structures reveals molecular effect of missense variants. *Proc. Natl. Acad. Sci. USA*, 117, 28201–28211. 33106425 10.1073/pnas.2002660117 PMC7668189

[61] Berman H.M. , Westbrook J., Feng Z. et al. (2000) The Protein Data Bank. *Nucleic Acids Res.*, 28, 235–242. 10592235 10.1093/nar/28.1.235 PMC102472

[62] Gupta A.B. , Verma R.K., Agarwal V. et al. (2012) MIPModDB: a central resource for the superfamily of major intrinsic proteins. *Nucleic Acids Res.*, 40, D362–D369. 22080560 10.1093/nar/gkr914 PMC3245135

[63] Jumpar J. , Evans R., Pritzel A. et al. (2021) Highly accurate protein structure prediction with AlphaFold. *Nature*, 596, 583–589. 34265844 10.1038/s41586-021-03819-2 PMC8371605

[64] Varadi M. , Anyango S., Deshpande M. et al. (2022) AlphaFold protein structure database: massively expanding the structural coverage of protein-sequence space with high-accuracy models. *Nucleic Acids Res.*, 50, D439–D444. 34791371 10.1093/nar/gkab1061 PMC8728224

[65] Pettersen E.F. , Goddard T.D., Huang C.C. et al. (2021) UCSF ChimeraX: structure visualization for researchers, educators, and developers. *Protein Sci.*, 30, 70–82. 32881101 10.1002/pro.3943 PMC7737788

[66] Ballesteros J.A. and Weinstein H. (1995) Integrated methods for the construction of three-dimensional models and computational probing of structure-function relations in G protein-coupled receptors. *Methods Neurosci.*, 25, 366–428.

[67] Gossweiner-Mohr N. , Siligan C., Pluhackova K. et al. (2022) The hidden intricacies of aquaporins: remarkable details in a common structural scaffold. *Small*, 18, Art. No. 2202056. 10.1002/smll.202202056 35802902

[68] Lomize M.A. , Pogozheva I.D., Joo H. et al. (2012) OPM database and PPM web server: resources for positioning of proteins in membranes. *Nucleic Acids Res.*, 40, D370–D376. 21890895 10.1093/nar/gkr703 PMC3245162

[69] de Groot B.L. , Frigato T., Helms V. et al. (2003) The mechanism of proton exclusion in the aquaporin-1 water channel. *J. Mol. Biol.*, 333, 279–293. 14529616 10.1016/j.jmb.2003.08.003

[70] Ilan B. , Tajkhorshid E., Schulten K. et al. (2004) The mechanism of proton exclusion in aquaporin channels. *Proteins Struct. Func. Bioinfo.*, 55, 223–228. 10.1002/prot.20038 15048815

[71] Wree D. , Wu B.H., Zeuthen T. et al. (2011) Requirement for asparagine in the aquaporin NPA sequence signature motifs for cation exclusion. *FEBS J.*, 278, 740–748. 21205205 10.1111/j.1742-4658.2010.07993.x

[72] Azad A.K. , Yoshikawa N., Ishikawa T. et al. (2021) Substitution of a single amino acid residue in the aromatic/arginine selectivity filter alters the transport profiles of tonoplast aquaporin homologs. *Biochim. Biophys. Acta, Biomembr.*, 1818, 1–11. 10.1016/j.bbamem.2011.09.014 21963407

[73] Oliva R. , Calamita G., Thornton J.M. et al. (2010) Electrostatics of aquaporin and aquaglyceroporin channels correlates with their transport selectivity. *Proc. Natl. Acad. Sci. USA*, 107, 4135–4140. 20147624 10.1073/pnas.0910632107 PMC2819975

[74] Kuriyan J. , Konforti B. and Wemmer D. (2012) *The Molecules of Life: Physical and Chemical Principles*. New York: Garland Science.

[75] Vajpai M. , Mukherjee M. and Sankararamakrishnan R. (2018) Cooperativity in plant plasma membrane intrinsic proteins (PIPs): mechanism of increased water transport in maize PIP1 channels in hetero-tetramers. *Sci. Rep.*, 8, Art. No. 12055. 10.1038/s41598-018-30257-4 PMC6089885 30104609

[76] Yoo Y.-J. , Lee H.K., Han W. et al. (2017) Interactions between transmembrane helices within monomers of the aquaporin AtPIP2;1 play a crucial role in tetramer formation. *Mol. Plant*, 9, 1004–1017. 10.1016/j.molp.2016.04.012 27142778

[77] Calvanese L. , D’Auria G., Vangone A. et al. (2018) Structural basis for mutations of human aquaporins associated to genetic diseases. *Int. J. Mol. Sci.*, 19, Art. No. 1577. 10.3390/ijms19061577 PMC6032259 29799470

[78] Kulandaisamy A. , Priya S.B., Sakthivel R. et al. (2018) MutHTP: mutations in human transmembrane proteins. *Bioinformatics*, 34, 2325–2326. 29401218 10.1093/bioinformatics/bty054

[79] Mottaz A. , David F.P.A., Veuthey A.-L. et al. (2010) Easy retrieval of single amino-acid polymorphisms and phenotype information using SwissVar. *Bioinformatics*, 26, 851–852. 20106818 10.1093/bioinformatics/btq028 PMC2832822

[80] The 1000 Genomes Project Consortium (2015) A global reference for human genetic variation. *Nature*, 526, 68–74. 26432245 10.1038/nature15393 PMC4750478

[81] Forbes S.A. , Beare D., Gunasekaran P. et al. (2015) COSMIC: exploring the world’s knowledge of somatic mutations in human cancer. *Nucleic Acids Res.*, 43, D805–D811. 25355519 10.1093/nar/gku1075 PMC4383913

[82] Stenson P.D. , Mort M., Ball E.V. et al. (2014) The Human Gene Mutation Database: building a comprehensive mutation repository for clinical and molecular genetics, diagnostic testing and personalized genome medicine. *Hum. Genet.*, 133, 1–9. 24077912 10.1007/s00439-013-1358-4 PMC3898141

[83] Lek M. , Karczewski K.J., Minikel E.V. et al. (2016) Analysis of protein-coding genetic variation in 60,706 humans. *Nature*, 536, 285–291. 27535533 10.1038/nature19057 PMC5018207

[84] Karczewski K.J. , Francioli L.C., Tiao G. et al. (2020) The mutational constraint spectrum quantified from variation in 141,456 humans. *Nature*, 581, 434–443. 32461654 10.1038/s41586-020-2308-7 PMC7334197

