# Supplementary material for: dbAQP-SNP: a database of missense single-nucleotide polymorphisms in human aquaporins
Source: Database (Oxford). 2023 Mar 13;2023:baad012. doi: 10.1093/database/baad012 (PMC10010469; doi:10.1093/database/baad012)
Supplement: baad012_Supp [file baad012_supp.zip › suppl_data/Sankar-hAQP-SNP-DB-paper-Supp-Info.docx]

**Supporting Information**

**dbAQP-SNP: A database of missense single nucleotide polymorphisms in human aquaporins**

**Rachana Dande^1^ and Ramasubbu Sankararamakrishnan^1,2,*^**

*^1^Department of Biological Sciences and Bioengineering, Indian Institute of Technology Kanpur, Kanpur 208016, Uttar Pradesh, India*

*^2^Mehta Family Centre for Engineering in Medicine, Indian Institute of Technology Kanpur, Kanpur 208026, Uttar Pradesh, India*

**Table S1:** Accession IDs of reference sequences of human aquaporin homologs

| Human AQP homolog | RefSeq ID of mRNA | RefSeq ID of protein |
| --- | --- | --- |
| AQP0 | NM 012064.3 | NP 036196.1 |
| AQP1 | NM 198098.3 | NP 932766.1 |
| AQP2 | NM 000486.5 | NP 000477.1 |
| AQP3 | NM 004925.4 | NP 004916.1 |
| AQP4 | NM 001650.6 | NP 001641.1 |
| AQP5 | NM 001651.3 | NP 001642.1 |
| AQP6 | NM 001652.3 | NP 001643.2 |
| AQP7 | NM 001170.2 | NP 001161.1 |
| AQP8 | NM 001169.2 | NP 001160.2 |
| AQP9 | NM 020980.4 | NP 066190.2 |
| AQP10 | NM 080429.2 | NP 536354.2 |
| AQP11 | NM 173039.2 | NP 766627.1 |
| AQP12 | NM 198998.2 | NP 945349.1 |

**Figure S1**


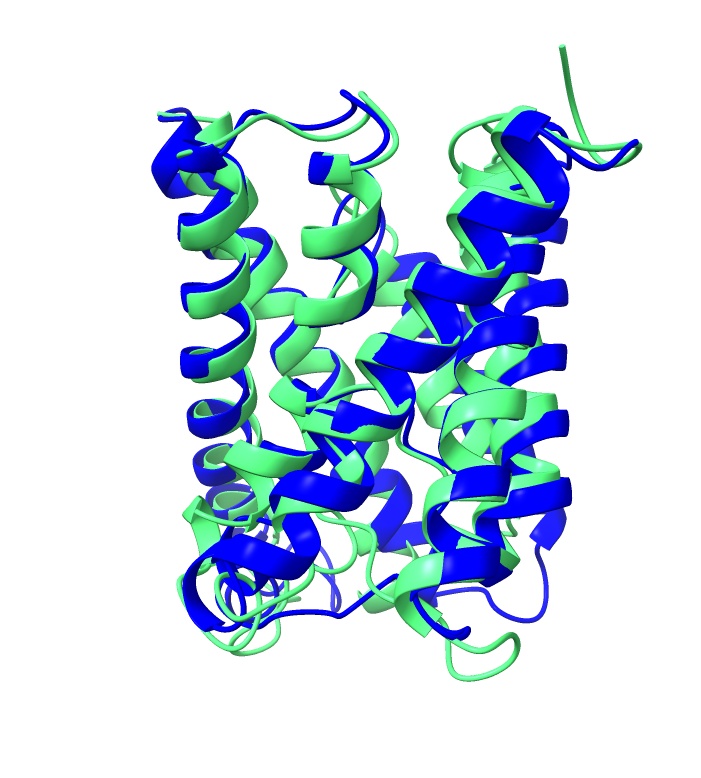

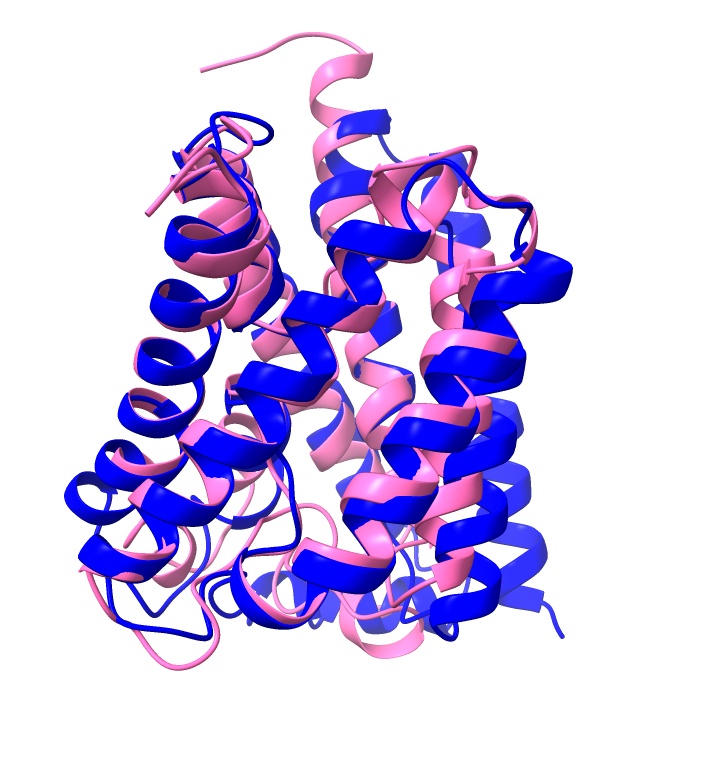


**Figure S1**: Superposition of models derived from AlphaFold (<https://alphafold.ebi.ac.uk>) [1,2] and downloaded from MIPModDB database (<http://bioinfo.iitk.ac.in/MIPModDB>) [3] shown for (left) AQP11 (right) AQP12 proteins. The structures predicted from AlphaFold are shown in blue for both AQP11 and AQP12. The structures downloaded from MIPModDB are shown in green and pink colors for AQP11 and AQP12 respectively. The molecular plots were rendered using ChimeraX (<https://www.cgl.ucsf.edu/chimerax/>) [4].

[1] Jumper et al., *Nature* **596**, 583-589 (2021)

[2] Varadi et al., *Nucleic Acids Res*. **50**, D439-D444 (2022)

[3] Gupta et al., *Nucleic Acids Res*. **40**, D362-D369 (2012)

[4] Pettersen et al., *Protein Sci*. **30**, 70-82 (2021)
